# Cost-Effectiveness of the START Hospital Addiction Consultation Service for Opioid Use Disorder Treatment

**DOI:** 10.1001/jamanetworkopen.2026.11324

**Published:** 2026-05-07

**Authors:** Adeyemi Okunogbe, Alexandra Peltz, Itai Danovitch, Allison J. Ober, Teryl K. Nuckols

**Affiliations:** 1 RAND, Arlington, Virginia; 2 Lucent Partners, Ibadan, Nigeria; 3 RAND, Santa Monica, California; 4 Cedars-Sinai Medical Center, Los Angeles, California

## Abstract

**Importance:**

People with opioid use disorder (OUD) are often hospitalized for emergent medical problems, but opioid use is seldom addressed during the inpatient stay. In a recent trial, patients randomized to Substance Use Treatment and Recovery Team (START), a hospital-based addiction consultation service, were more likely to initiate medication for opioid use disorder and be linked to OUD-focused follow-up care compared with patients receiving usual OUD care, which was at the discretion of the primary team.

**Objective:**

To evaluate the incremental cost-effectiveness of START from the health sector and limited societal perspectives relative to usual care.

**Design, Setting, and Participants:**

This economic evaluation was an incremental cost-effectiveness analysis based on the START trial, which was conducted at 3 major academic medical centers. START participants were adults with a probable OUD diagnosis documented during an inpatient hospitalization from November 2021 to September 2023. This economic evaluation used trial participant–level cost and health outcomes data, supplemented with published data.

**Exposures:**

Two strategies were compared: (1) START and (2) usual care.

**Main Outcomes and Measures:**

Costs, quality-adjusted life year (QALYs), and incremental cost-effectiveness ratios (ICERs). ICERs were estimated using a Markov model over a 12-month horizon, with deterministic and probabilistic sensitivity analyses. ICERs were expressed as cost per QALY gained, with a willingness-to-pay threshold of USD $150 000/QALY.

**Results:**

A total of 325 participants were randomized to the START (164 [50.5%]) or usual care (161 [49.5%]); 213 were male (66%) and the median (IQR) age was 41 (32-50) years. START implementation costs were $640 per patient (personnel, $602; training and onboarding, $38). Compared with usual care, START was associated with an incremental cost of $162 (95% UI, −$93 to $179) and a gain in QALYs of 0.0103 (95% UI, 0.0102 to 0.0106) per person from a health sector perspective, leading to an ICER of $15 750 (95% UI, $8742 to $17 034) per QALY gained. The ICER was $20 921 (95% UI, $13 747 to $22 190) per QALY gained from a limited societal perspective. Sensitivity analyses demonstrated that health care expenditures and intervention effectiveness were the strongest drivers of cost-effectiveness.

**Conclusions and Relevance:**

In this trial-based economic evaluation, START was a cost-effective approach for addressing opioid use disorder in the inpatient setting by increasing the initiation of medication for OUD and linkage to OUD-focused care after discharge.

## Key Points

**Question:**

Is a hospital-based addiction consultation service (ACS) cost-effective for increasing the initiation of US Food and Drug Administration–approved medication for opioid use disorder in the hospital and linking patients to follow-up care after discharge?

**Findings:**

In this economic evaluation of 325 patients, a hospital-based ACS was cost-effective compared with usual care. From a health-sector perspective, the ACS was associated with an incremental cost of $162 and 0.0103 quality-adjusted life-year (QALY) gained per person, leading to an incremental cost-effectiveness ratio of $15 750/QALY gained.

**Meaning:**

These findings suggest that the hospital-based ACS was a cost-effective way to improve care for hospitalized patients with opioid use disorder.

## Teaser

This economic evaluation examines the cost-effectiveness of a hospital-based addiction consultation service from both health sector and limited societal perspectives.

## Introduction

The prevalence of opioid use disorder (OUD) in the US climbed rapidly for more than 2 decades, affecting 5.7 million people and causing almost 80 000 deaths in 2023.^1,2,3^ US Food and Drug Administration (FDA)–approved medications for opioid use disorder (MOUD), which include buprenorphine, methadone, and extended-release naltrexone, can substantially reduce cravings, overdoses, and/or mortality.^1,4,5,6^ However, MOUD is markedly underused,^4,5^ with only 18% of people receiving it.^3^

People with OUD are hospitalized frequently for acute medical conditions, both related to and unrelated to substance use,^7^ with an average rate of about 24% per year.^8^ From 2005 to 2020, opioid-related hospitalizations rose from 137 to 250 per 100 000 population,^7^ accounting for 3.4% of all hospitalizations in metropolitan areas. Yet, few inpatients receive MOUD, even after overdose,^9^ resulting in high posthospitalization mortality rates^10^ and profound costs at the patient, clinician, health system, and societal levels.^10,11,12,13^

To leverage hospitalization as an opportunity to increase use of MOUD, addiction consultation services (ACS) are a promising approach. Prior studies have shown that ACS can increase postdischarge engagement in addiction treatment and reduce addiction severity.^14^ In a recent randomized clinical trial, our team demonstrated that a hospital-based ACS intervention, called the Substance Use Treatment and Recovery Team (START), significantly increased the rates at which inpatients initiated FDA-approved MOUD during hospitalization and successfully linked to OUD-focused follow-up care after discharge, relative to usual care.^15^ START consists of an addiction medicine specialist (AMS) and a care manager (CM) who use evidence-based tools and resources to reduce barriers to MOUD initiation and follow-up care.^16^

However, the cost of ACS programs like START is a key factor in hospitals’ adoption and implementation decisions, underscoring the need for robust cost-effectiveness analyses. Yet, little evidence exists on the cost-effectiveness of ACS models designed to initiate MOUD in the inpatient setting.^17,18,19,20,21^ This economic evaluation seeks to assess the START intervention’s implementation cost, incremental net cost, and incremental cost-effectiveness ratio (ICER) relative to usual care.

## Methods

This economic evaluation was a secondary, ad hoc analysis of the START trial (NCT05086796) and was reported in accordance with the Consolidated Health Economic Evaluation Reporting Standards (CHEERS) reporting guideline (eTable 1 in Supplement 1).^22^ The trial documents, protocol, and statistical analysis plan can be found in Ober et al^16^ and Danovitch et al^23^; prior studies have been published about the design and effectiveness of START.^15,24,25^

### Setting and Participants

The START trial was conducted at 3 large academic medical centers: Cedars-Sinai Medical Center in Los Angeles, California; the University of New Mexico Hospital in Albuquerque, New Mexico; and Baystate Medical Center in Springfield, Massachusetts. The Cedars-Sinai Medical Center institutional review board approved the study. All participants provided informed consent.

The trial was an open-label, parallel-group, superiority trial that randomized individual patients to START or usual care in a 1:1 ratio. Participants were inpatients from November 2021 to September 2023, were age 18 years or older, had a probable OUD diagnosis (based on enrollment survey), were not already receiving MOUD, spoke English or Spanish, and were not in hospice. We assessed the initiation of MOUD via medical record review and linkage to follow-up care via telephone interviews conducted 30 to 60 days after discharge.

### Comparators

The trial and economic evaluation compared START and usual care. In START, an addiction medicine specialist and care manager worked together to deliver a tailored intervention based on motivational interviewing and addiction-focused discharge planning. The START team performed diagnostic assessments, made clinical recommendations, assisted with treatment plan implementation, established OUD-focused discharge plans, facilitated linkage to treatment after discharge, and provided follow-up telephone calls for 1 month. Usual OUD care was consistent with standard practice at each hospital, which could include assessment, patient education, consultation, treatment initiation, and referral to postdischarge care, and was at the discretion of the primary clinical team.^15,16^

### Economic Evaluation Model Overview

We developed a state-transition Markov model to compare START vs usual care from the health sector and limited societal perspectives, as recommended by the Second Panel on Cost-Effectiveness in Health and Medicine (eTable 2 in Supplement 1).^26^ Model outcomes were incremental net cost (2023 US dollars), incremental effectiveness (quality-adjusted life years [QALYs]), and the ICER from each perspective.

By applying a Markov modeling approach, we were able to simulate transitions across treatment states that people with OUD may experience over time, thereby providing a realistic estimate of intervention impacts. We chose a 12-month time horizon because START had a short follow-up period and because a shorter time horizon reflected a conservative estimate of cost-effectiveness.

### Health States

We modeled transitions among 4 health states (eFigure 1 in Supplement 1): (1) untreated OUD (not taking MOUD at hospital admission), (2) MOUD-initiated (MOUD initiated during hospitalization), (3) MOUD-sustained (MOUD continued after initiation), and (4) death (during or after hospitalization). The model had a cycle length of 1 month. Consistent with trial eligibility criteria, patients entered the model in the untreated OUD state (eFigure 2 in Supplement 1). After the first cycle, patients could remain in this state or transition to any other state. Patients in the MOUD-initiated state could transition to any other state. Patients in the MOUD-sustained state could remain there, revert to untreated OUD, or transition to death. Death was an absorbing state, meaning no further transitions were possible (eMethods in Supplement 1). For each health state, we assigned costs and a quality-of-life weight, based on data from the START clinical trial, published literature, and publicly available databases (Table 1).

**Table 1.** Selected Model Parameters

Parameter	Baseline (range)		PSA distribution	Source
	START	Usual care		
**Clinical parameters**				
Probability of transitioning between health states, per 30 d, %				
Untreated OUD to MOUD-initiated	57.3 (39.7-65.0)	26.7 (19.8-33.6)	PERT	START Trial: Ober et al,^15^ 2025^a^
MOUD-initiated to MOUD-sustained	71.4 (60.6-82.3)	70.8 (51.2-90.4)	Uniform	START Trial: Ober et al,^15^ 2025^a^
MOUD-sustained to MOUD-sustained	54.9 (46.7-63.2)	54.3 (46.1-62.4)	Uniform	START Trial: Ober et al,^15,27^ 2025^a^; Biondi et al,^15,27^ 2022
Transition to death from				
Untreated OUD	0.41 (0.29-0.52)	0.41 (0.29-0.52)	β	Ma,^28^ 2019
MOUD-initiated	0.12 (0.02-0.22)	0.12 (0.02-0.22)	β	Ma,^28^ 2019
MOUD-sustained	0.05 (0.04-0.05)	0.05 (0.04-0.05)	β	Ma,^28^ 2019
Health state utilities (QALY, monthly)			β	
Untreated OUD	0.047 (0.044-0.050)	0.047 (0.044-0.050)	β	Wittenberg et al,^29^ 2016
MOUD-initiated	0.054 (0.051-0.057)	0.054 (0.051-0.057)	β	Wittenberg et al,^29^ 2016
MOUD-sustained	0.064 (0.061-0.066)	0.064 (0.061-0.066)	β	Wittenberg et al,^29^ 2016
**Cost parameters**				
Total health care expenditures per mo, $				
Untreated OUD	4184 (2092-6276)	4184 (2092-6276)	γ	Larochelle et al,^30^ 2020
MOUD-initiated	4149 (2075-6224)	4149 (2075-6224)	γ	Larochelle et al,^30^ 2020
MOUD-sustained	2886 (1443-4329)	2886 (1443-4329)	γ	Larochelle et al,^30^ 2020
Informal health care costs per mo, $			γ	
Patient time costs	115 (58-173)	115 (58-173)	γ	BLS,^31^ 2022
Transportation costs	55 (27-82)	55 (27-82)	γ	Kleinman,^32^ 2020
Indirect costs per mo, $			γ	
Absenteeism costs when untreated	50 (25-74)	50 (25-74)	γ	Henke et al,^33^ 2020
Absenteeism costs in treatment	7 (4-11)	7 (4-11)	γ	Henke et al,^33^ 2020
Lost earnings from mortality	4756 (2378-7134)	4756 (2378-7134)	γ	BLS,^31^ 2022

Abbreviations: BLS, Bureau of Labor Statistics; MOUD, medications for opioid use disorder; OUD, opioid use disorder; PERT, program evaluation and review technique; PSA, probabilistic sensitivity analyses; QALY, quality-adjusted life-years; START, Substance Use Treatment and Recovery Team.

^a^ Data from START Trial.

### Transition Probabilities

We estimated transition probabilities for both the START and usual care groups (Table 1).^15,27,28,29,30,31,32,33^ For the probability of transitioning from the untreated OUD to MOUD-initiated state, the first Markov cycle relied on trial data for each group, specifically, the observed probabilities of initiating MOUD during hospitalization. In subsequent cycles, we used the same probability for both groups, based on a published estimate for community-dwelling US adults needing OUD treatment (eTable 3 in Supplement 1).^1^

For the probability of moving from MOUD-initiated state to MOUD-sustained state, we relied on trial data for each group (specifically, the observed probabilities of continuing MOUD through the 30-day follow-up period). For the probability of remaining in the MOUD-sustained state, we used published estimates on longer-term retention in MOUD treatment.^27^ The probabilities of transitioning from the other health states to death were based on published estimates of crude mortality rate among adults with OUD, among those who initiate MOUD, and among those who sustain MOUD.^28^

### Costs

For the health sector perspective, costs included START implementation costs and health outcome-related costs. For the limited societal perspective, we included the same costs plus informal health care costs and productivity losses from absenteeism (missed time from work due to OUD), presenteeism (reduced productivity at work due to OUD), and premature mortality. All costs were converted to 2023 US dollars using the gross domestic product price index.^34,35^

#### Implementation Costs

These costs only applied to participants randomized to the START intervention and to the first Markov cycle. Implementation costs comprised personnel costs (time spent delivering START by the START team) and onboarding and training costs (the time spent training and onboarding the START Team on START intervention components). Using a patient registry, the AMS and CM each recorded time spent per activity for each patient during the inpatient stay and over 4 weeks after discharge. Personnel time included time spent interacting with patients, linking patients to outpatient care after discharge, making follow-up calls within 4 weeks postdischarge, and performing administrative tasks. Onboarding and training time included both the trainer’s and trainee’s time. We calculated the personnel costs and training and onboarding costs by multiplying the time spent by the hourly wage with fringe benefits, using data from the Bureau of Labor Statistics.^31^ We assumed that intervention costs in the usual care group were zero, even though a few patients might have had consultations for addiction care and case management assistance.

#### Health Outcome–Related Costs

For each living health state, total monthly health care expenditures were derived from a study reporting costs before, during, and after MOUD initiation among insured adults with OUD.^30^ The study estimated total aggregate health care expenditures related to OUD and other health conditions, including both health plan and patient out-of-pocket expenditures. Expenditures were based on adjudicated claims that included medical, surgical, behavioral health, laboratory, durable medical equipment, and pharmacy claims in all inpatient and outpatient care settings. We used the same cost estimates in the intervention and usual care groups.

#### Informal Health Care Costs

We included transportation and patient time costs in the limited societal perspective. Transportation costs were calculated by multiplying the median distance to an opioid treatment program, the estimated number of visits over 12 months, and the standard reimbursement mileage rate (eTable 4 in Supplement 1).^32,36^ The estimated number of visits was calculated from an assumed treatment schedule based on national guidelines.^37^ Patient time costs were calculated by multiplying the estimated time spent per visit, the number of visits over a 12-month period, and the US median hourly wage (eTable 5 in Supplement 1).

#### Productivity Losses and Premature Mortality Costs

To estimate productivity losses due to absenteeism and presenteeism, respectively, we multiplied days of missed work and reduced productivity at work in a month (due to illness among those with OUD) by the US median daily wage (eTable 6 in Supplement 1).^31,33^ Premature mortality costs were estimated as the monthly lost earnings due to premature death, computed by multiplying weekly wages^31^ by the mean number of workweeks per month.

### Health Utilities

For each health state (untreated OUD, MOUD-initiated, and MOUD-sustained), we used published estimates of health-related quality of life for opioid misuse and treatment states in the US,^29^ which were based on the standard gamble method.^38^ Estimates were divided by 12 to convert to monthly utilities (Table 1). We used the same values in START and usual care groups.

### Statistical Analysis

The primary economic outcome, ICER, was calculated as the difference in total costs (depending on the economic perspective) between the START and usual care groups, divided by the difference in QALYs between both groups. We used $150 000/QALY as the cost-effectiveness threshold, consistent with theoretical and empirical evidence in the US.^39,40,41^ Given the short-term horizon of 1 year, discounting was not performed.

Evidence consistently shows that individuals with prior exposure to MOUD are more likely to initiate or reinitiate MOUD than those who have never received it.^42,43,44^ To explore this, we conducted a scenario analysis where we assumed that the initiation rate among START patients (who did not initiate MOUD during the first cycle) could be as much as 48% higher than usual care patients postdischarge, based on START trial data (comparing the number of those who initiated MOUD during the 1-month follow-up period in the 2 trial groups). Hence, in months 2 to 12, we weighted the community-dwelling MOUD initiation rate used in the base-case analysis upwards by 48% for those in the START group.

#### Sensitivity Analyses

We conducted a series of 1-way sensitivity analyses to examine the implications of uncertainty in individual model parameters and to understand the key drivers of our results. Parameters tested included START implementation costs, transition probabilities, and health care costs for each living health state. We varied each model parameter between the lower and upper bound values of feasible ranges. We defined probabilistic distributions for model parameters and ran 5000 Markov Chain Monte Carlo simulations, each using parameter values randomly selected from the distributions (Table 1 and eMethods in Supplement 1).

Two-tailed tests were used at *P* <  .05 level of significance to examine the statistical difference between participants who took part in follow-up interview compared with those who did not. All analyses were performed between November 2024 and August 2025 using Stata version 15 (StataCorp) and TreeAge Pro version 2025 R1.1 (TreeAge Software).

## Results

As previously reported,^15^ participants had a median (IQR) age of 41 (32-50) years and 213 (65.5%) identified as male. This study included 325 participants who were randomized to the START (164 [50.5%]) or usual care (161 [49.5%]) groups; 229 participants had a 30-day postdischarge follow-up interview.^15^

### Base Case Analysis

START implementation cost was $640 per patient (personnel, $602; training and onboarding, $38) (Table 2). From the health sector perspective, the net cost for the START group was $50 206 with 0.646 (95% UI, 0.646 to 0.647) QALYs per patient. For the usual care group, the net cost was $50 044 with 0.636 (95% UI, 0.635 to 0.636) QALYs per patient. Compared with usual care, START resulted in an incremental net cost of $162 (95% UI, −$93 to $179) and a gain in QALYs of 0.0103 (95% UI, 0.0102 to 0.0106) per person, yielding an ICER of $15 750 (95% UI, $8742 to $17 034) per QALY gained. From the limited societal perspective, the ICER was $20 921 (95% UI, $13 747 to $22 190) per QALY gained (Table 3).

**Table 2.** Implementation Costs per Patient for the Substance Use Treatment and Recovery Team (START) Intervention (2023 US Dollars)^a^

Personnel	Time, h^b^	Estimated hourly wage, $^c^	Cost per patient, $
**Training costs**			
START Trainer	15	165.75	15.16
AMS			
Site 1	5	133.24	4.06
Site 2	5	209.08	6.37
Site 3	5	154.93	4.72
CM			
Site 1	5	82.59	2.52
Site 2	5	89.00	2.71
Site 3	5	88.12	2.69
Total cost per patient	NA	NA	38.24
**Personnel costs**			
AMS			
All sites	1.73	165.75	286.75
Site 1	1.66	133.24	221.71
Site 2	2.25	209.08	470.42
Site 3	1.58	154.93	244.18
CM			
All sites	3.64	86.57	315.11
Site 1	2.60	82.59	215.05
Site 2	4.72	89.00	420.30
Site 3	3.54	88.12	312.30
Total cost per patient	NA	NA	601.86

Abbreviations: AMS, addiction medicine specialist; CM, care manager; NA, not applicable.

^a^ Training costs are the time costs of training and on-boarding the START Team. Personnel costs are the time costs of delivering START.

^b^ Defined according to Ober et al.^15^

^c^ Defined according to the US Bureau of Labor Statistics.^45^

**Table 3.** Incremental Cost-Effectiveness of the Substance Use Treatment and Recovery Team (START) Intervention, Health Sector, and Limited Societal Perspectives (2023 US Dollars)

Perspectives	Net cost per patient (95% UI), $	Incremental net cost per patient (95% UI)	QALYs per patient (95% UI)	Incremental QALYs per patient (95% UI)	Incremental cost-effectiveness ratio per QALY (95% UI), $
**Health sector**					
START	50 206 (50 112 to 50 573)	162 (−93 to 179)	0.646 (0.646 to 0.647)	0.0103 (0.0102 to 0.106)	15 750 (8742 to 17 034)
Usual care	50 044 (49 955 to 50 457)		0.636 (0.635 to 0.636)		
**Limited societal**					
START	53 021 (52 807 to 53 265)	215 (142 to 225)	0.646 (0.646 to 0.647)	0.0103 (0.0103 to 0.0107)	20 921 (13 747 to 22 190)
Usual care	52 806 (52 605 to 53 100)		0.636 (0.635 to 0.636)		

Abbreviations: QALYs, quality-adjusted life-years; UI, uncertainty interval.

In the scenario analysis where the probability of initiating MOUD in subsequent cycles was 48% higher than in usual care, START became cost-saving from both health-sector and limited societal perspectives. The average cost of START was lower than usual care by $432 (health sector) and $237 (limited societal) (eTable 7 and eFigures 3 and 4 in Supplement 1).

### Sensitivity Analyses

**Figure 1.**
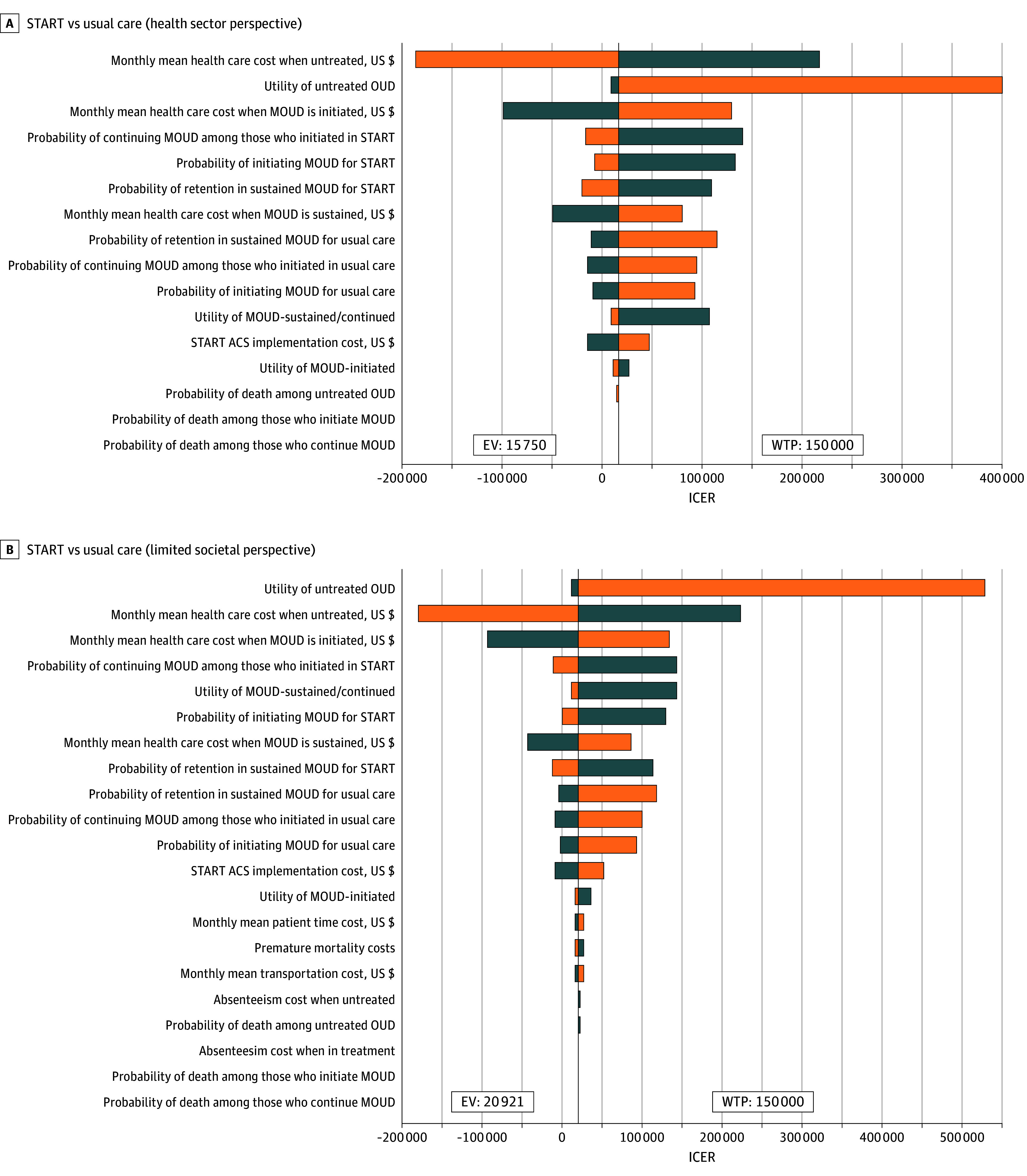
Tornado Diagrams for Deterministic Sensitivity Analyses of Incremental Cost-Effectiveness Ratios (ICERs) From the Health Sector and Limited Societal Perspectives Model parameters were varied between lower and upper bound of their feasible ranges (±50%, ±15%, and ±30% of the base case value for costs, transition probabilities, and utilities, respectively). ACS indicates addiction consultation service; EV, expected value; OUD, opioid use disorder; MOUD, medication for OUD; START, Substance Use Treatment and Recovery Team; WTP, willingness to pay.

**Figure 2.**
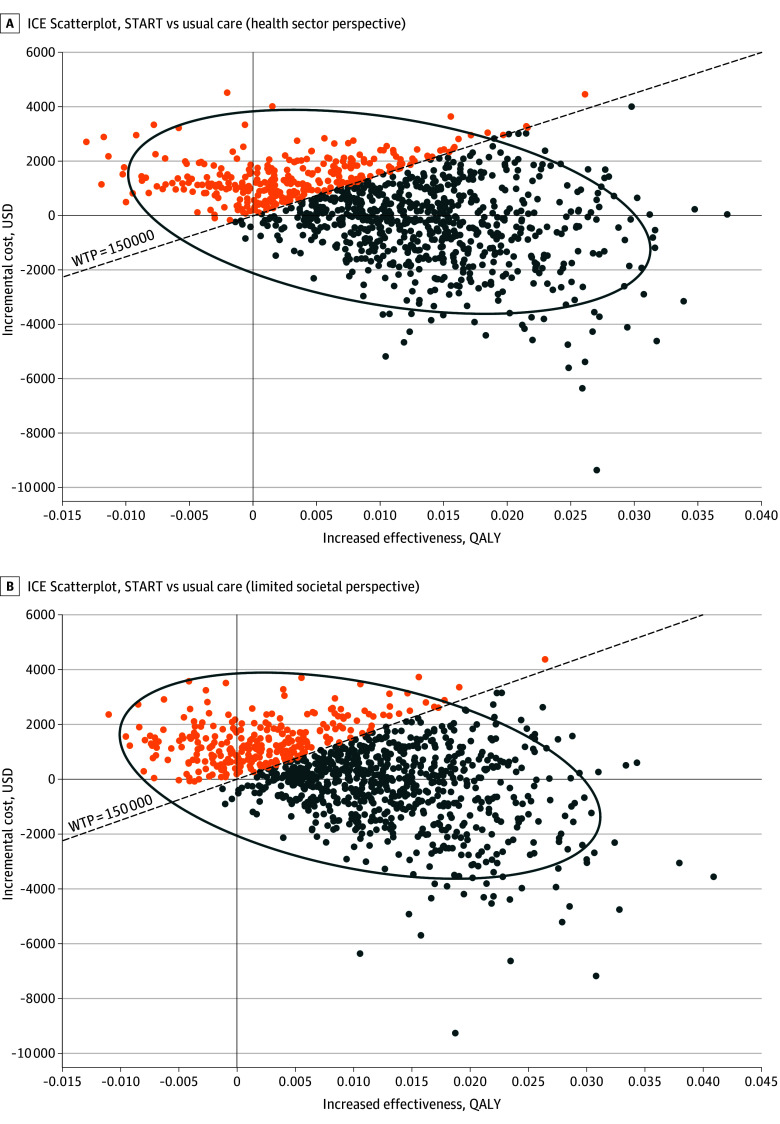
Probabilistic Sensitivity Analysis Scatter Plot: Incremental Cost-Effectiveness (ICE) From the Health Sector and Limited Societal Perspectives (Based on 5000 Simulations) START was the optimal strategy in 71.80% (health sector perspective) and 70.52% (limited societal perspective) of simulations. QALY indicates quality-adjusted life years; START, Substance Use Treatment and Recovery Team; USD, US dollars; WTP, willingness to pay.

From deterministic analyses, key parameters driving results included the utility of untreated OUD health state, health care expenditures associated with the untreated and MOUD-initiated health states, and the probabilities of initiating and sustaining MOUD in the START group (Figure 1 and eTables 8 and 9 in Supplement 1). In probabilistic sensitivity analyses, START was the optimal strategy in 71.80% (health sector perspective) and 70.52% (limited societal perspective) of simulations (Figure 2).

## Discussion

This economic evaluation examined the cost-effectiveness of the START intervention, which we previously found to be effective at increasing the initiation of MOUD during hospitalization and successfully linking patients to OUD-focused follow-up care after discharge, relative to usual care.^15^ START was relatively inexpensive to implement on a per-patient basis: $640 in 2023 US dollars. Notably, START was cost-effective from the health sector perspective within just 1 year, with an ICER of $15 750/QALY gained, compared to usual care. This ICER is less than one-fifth of the US per-capita gross domestic product ($82 769 in 2023), an accepted threshold for the willingness to pay for health.^46^

The cost-effectiveness of START can largely be attributed to the higher health care expenditures among people with untreated OUD, compared with those on sustained MOUD treatment. Over a 12-month horizon, the health care cost reduction substantially offset START implementation costs. Considering that implementation costs may decline over time as more people are served, the realized offset could even become greater. These findings support the value of addressing OUD during hospitalization, regardless of whether the admission is directly associated with substance use.

Our sensitivity analyses revealed that the most influential parameters were the health care costs associated with each health state in the model and the effectiveness of START in facilitating MOUD initiation. The implementation cost of START was among the least influential parameters.

To our knowledge, this is the first study to evaluate the cost-effectiveness of an ACS for hospital-based MOUD initiation in an inpatient setting based on a randomized clinical trial. Our results were comparable with other studies that have explored the cost-effectiveness of MOUD initiation in similar settings. Barocas et al^19^ in a microsimulation study estimated the cost-effectiveness of $14 300 when MOUD is combined with addiction consult services compared with the status quo (detoxification for opioids, no addiction consult service) in hospital-based settings. For MOUD initiation at treatment facility-based medically managed withdrawal programs, Savinkina et al^47^ estimated an ICER of $56 000/QALY from a health sector perspective over 10 years. For a community-based program that involved expanded access to MOUD, Fairley et al^17^ demonstrated cost savings of $25 000 to $105 000 to society per person over a lifetime horizon. Krebs et al^48^ demonstrated lifetime cost savings of $78 257 per person from a societal perspective if immediate access to MOUD was provided in California’s publicly funded treatment facilities. Our analysis likely revealed cost-effectiveness rather than cost savings due to our use of a shorter time horizon, omission of broader societal benefits, and conservative assumption that linkage to follow-up care alone would not increase initiation of MOUD after hospital discharge. The ICER estimate from the limited societal perspective was higher than that from the health sector perspective. This was because over the short 1-year horizon, the reductions in premature mortality costs were not large enough to offset the additional formal and informal health care costs associated with increased MOUD initiation. When the time horizon was extended beyond 18 months, the ICER estimate from the limited societal perspective became lower than that of the health-sector perspective (eFigure 5 in Supplement 1).

In deciding whether to implement new evidence-based interventions, hospitals are often concerned about implementation costs.^49^ However, for START, about half of the implementation costs consisted of billable services by the AMS, so most hospitals could reasonably implement START without a substantial budgetary impact. In an integrated health system, the financial implications become even more favorable because implementation costs would be offset by patients having lower health care expenditures with sustained MOUD treatment. For policymakers considering broader societal impacts over a longer-term horizon, the high volume of hospitalized patients with untreated OUD, their high mortality rates, and the addition of societal costs, including productivity and informal health care costs, make this intervention highly cost-effective. If hospitals’ uptake of ACS for OUD remains limited, federal and commercial payers could consider value-based payment models that incentivize hospitals to adopt such services, paralleling existing policies that focus on common presenting medical conditions like pneumonia, myocardial infarction, and heart failure.

### Strengths and Limitations

A major strength of this study was using data from the START randomized trial. However, deriving effectiveness data from large academic hospitals could limit generalizability to smaller institutions. Retention of trial participants at the 30-day follow-up interview was 70% overall, and better for START than usual care (76.2% vs 64.6%).^15^ However, these limitations likely had modest effects on the ICER, based on the deterministic sensitivity analyses of the probability of MOUD retention. We drew mortality rates and health care expenditure estimates from published literature that may not accurately reflect our study population, but are more generalizable.^30,45,50,51,52^ Because use of the START intervention was entirely under the control of the investigators, true crossovers did not occur. Nonetheless, usual care for control patients may have been indirectly influenced by the study, potentially improving OUD care more broadly and thereby attenuating observed differences in effectiveness and cost-effectiveness between groups. Important societal costs, such as crime, were not captured in this analysis. However, our results are consistent with other comparable studies, and overarching findings were robust to or even more favorable under multiple sensitivity analyses.

## Conclusions

In this economic evaluation, we found evidence that an addiction consultation service that initiates MOUD among adults who are hospitalized with OUD and facilitates linkage to MOUD-related follow-up care improved health outcomes for people with OUD and was cost-effective. Future work will examine the cost-effectiveness of addiction consultation services for OUD over longer time horizons, incorporate broader societal costs, and explore their applicability to other substance use disorders.

## Notes

Supplement 1.
eMethods
**eTable 1.** CHEERS Checklist (Consolidated Health Economic Evaluation Reporting System) **eTable 2.** Impact Inventory Analysis **eTable 3.** Transition Probabilities for Initiating and Sustaining MOUD by Markov Cycle **eTable 4.** Transportation Costs **eTable 5.** Patient Time Costs **eTable 6.** Productivity Losses due to Absenteeism and Presenteeism Associated with OUD **eTable 7.** Incremental Cost-Effectiveness of the Substance Use Treatment and Recovery Team” (START) Intervention (2023 US Dollars)-- Key Scenario **eTable 8.** Deterministic Sensitivity Analyses (Health Sector Perspective) **eTable 9.** Deterministic Sensitivity Analyses (Limited Societal Perspective) **eFigure 1.** State Transition Diagram **eFigure 2.** Markov Model Diagram **eFigure 3.** Probabilistic Sensitivity Analysis Scatter Plot: Incremental Cost- Effectiveness from the Health Sector Perspective (based on 5,000 simulations)- Key Scenario **eFigure 4.** Probabilistic Sensitivity Analysis Scatter Plot: Incremental Cost- Effectiveness from the Limited Societal Perspective (based on 5,000 simulations) - Key Scenario **eFigure 5.** ICER Estimates by Time Horizon
eReferences

Supplement 2.
Data Sharing Statement
