# Error in Figure 2A

**DOI:** 10.1001/jamanetworkopen.2026.20725

**Published:** 2026-05-26

In the Original Investigation titled “Cost-Effectiveness of the START Hospital Addiction Consultation Service for Opioid Use Disorder Treatment,”^1^ published May 7, 2026, the graph in Figure 2B was duplicated in Figure 2A. The article was revised to include the correct graph for Figure 2A. This article has been corrected.^1^

## References

[1] Okunogbe A, Peltz A, Danovitch I, Ober AJ, Nuckols TK. Cost-effectiveness of the START hospital addiction consultation service for opioid use disorder treatment. JAMA Netw Open. 2026;9(5):e2611324. doi:10.1001/jamanetworkopen.2026.11324 42095700 PMC13153993

