# Supplementary material for: Cost-Effectiveness of the START Hospital Addiction Consultation Service for Opioid Use Disorder Treatment
Source: JAMA Netw Open. 2026 May 7;9(5):e2611324. doi: 10.1001/jamanetworkopen.2026.11324 (PMC13153993; doi:10.1001/jamanetworkopen.2026.11324)
Supplement: Supplement 1. — eMethods eTable 1. CHEERS Checklist (Consolidated Health Economic Evaluation Reporting System) eTable 2. Impact Inventory Analysis eTable 3. Transition Probabilities for Initiating and Sustaining MOUD by Markov Cycle eTable 4. Transportation Costs eTable 5. Patient Time Costs eTable 6. Productivity Losses due to Absenteeism and Presenteeism Associated with OUD eTable 7. Incremental Cost-Effectiveness of the Substance Use Treatment and Recovery Team” (START) Intervention (2023 US Dollars)-- Key Scenario eTable 8. Deterministic Sensitivity Analyses (Health Sector Perspective) eTable 9. Deterministic Sensitivity Analyses (Limited Societal Perspective) eFigure 1. State Transition Diagram eFigure 2. Markov Model Diagram eFigure 3. Probabilistic Sensitivity Analysis Scatter Plot: Incremental Cost- Effectiveness from the Health Sector Perspective (based on 5,000 simulations)- Key Scenario eFigure 4. Probabilistic Sensitivity Analysis Scatter Plot: Incremental Cost- Effectiveness from the Limited Societal Perspective (based on 5,000 simulations) - Key Scenario eFigure 5. ICER Estimates by Time Horizon eReferences [file jamanetwopen-e2611324-s001.pdf]

Supplemental Online Content

Cost-Effectiveness of the START Hospital Addiction Consultation Service for Opioid Use Disorder Treatment

Supplemental Online Content

Okunogbe A, Peltz A, Danovitch I, Ober AJ, Nuckols T. Cost-effectiveness of the START hospital addiction consultation service for opioid use disorder treatment. *JAMA Netw Open*. 2026;9(5):e2611324. doi:10.1001/jamanetworkopen.2026.11324

eMethods
eTable 1. CHEERS Checklist (Consolidated Health Economic Evaluation Reporting System)
eTable 2. Impact Inventory Analysis
eTable 3. Transition Probabilities for Initiating and Sustaining MOUD by Markov Cycle
eTable 4. Transportation Costs
eTable 5. Patient Time Costs
eTable 6. Productivity Losses due to Absenteeism and Presenteeism Associated with OUD
eTable 7. Incremental Cost-Effectiveness of the Substance Use Treatment and Recovery Team" (START) Intervention (2023 US Dollars)-- Key Scenario
eTable 8. Deterministic Sensitivity Analyses (Health Sector Perspective)
eTable 9. Deterministic Sensitivity Analyses (Limited Societal Perspective)
eFigure 1. State Transition Diagram
eFigure 2. Markov Model Diagram
eFigure 3. Probabilistic Sensitivity Analysis Scatter Plot: Incremental Cost- Effectiveness from the Health Sector Perspective (based on 5,000 simulations)- Key Scenario
eFigure 4. Probabilistic Sensitivity Analysis Scatter Plot: Incremental Cost- Effectiveness from the Limited Societal Perspective (based on 5,000 simulations) - Key Scenario
eFigure 5. ICER Estimates by Time Horizon
eReferences

This supplemental material has been provided by the authors to give readers additional information about their work.

© 2026 Okunogbe A et al. *JAMA Network Open*.

eMethods

Technical Details
We implemented the model in TreeAge Pro Version 2025 R1.1 (https://www.treeage.com)[1], and estimated parameter values using STATA (https://www.stata.com)[2]. We calculated the incremental cost effectiveness ratios (ICERs) from the health sector perspective and a limited societal perspective. We calculated ICERs, conducted deterministic sensitivity analyses and probabilistic sensitivity analyses using Markov Chain Monte Carlo simulations in TreeAge Pro.

Model Overview and Structure
We developed a state-transition Markov model to evaluate the cost-effectiveness of the START ACS[3] compared to usual care over a 12-month period, with a cycle length of one month. All patients enter the model in the Untreated opioid use disorder (OUD) state, consistent with the START trial's eligibility criteria.

Health States
The model considered transitions among four health states (eFigure 1 & 2): The health states are (1) Untreated OUD (not on medications for OUD [MOUD] at hospital admission), (2) MOUD-Initiated (initiated MOUD during hospitalization), (3) MOUD-Sustained (continued MOUD after discharge), and (4) Death (during or after hospitalization). The model had a cycle length of one month. The first Markov cycle reflected events occurring from hospital admission through discharge; for the intervention arm, this represented START. After the first cycle (where all patients were in Untreated OUD state), patients could either remain in the Untreated OUD state or transition to any of the other health states. The second Markov cycle approximated events occurring within the first 30 days after hospitalization i.e., until trial completion. The third and subsequent Markov cycles reflected events after the trial. Patients in the MOUD-Initiated state could transition to any other state. Patients in the MOUD-Sustained state could remain there, revert to Untreated OUD, or transition to Death. Death was an absorbing state, meaning no further transitions were possible.

Transition probabilities
Transition probabilities between health states were derived from the START clinical trial data and published literature (Table 1, eTable 1). The transitions captured under each health state are as follows:

From Untreated OUD state: All patients begin in this health state. Subsequently, subjects can remain in this health state, move to the MOUD-Initiated state (i.e. initiate MOUD during hospitalization) or die. For moving to MOID-Initiated in the first Markov cycle, we derived the associated probabilities from the proportions of patients in the START treatment and control groups that initiated MOUD during hospitalization. In subsequent cycles, we used the MOUD initiation rate among community dwelling U.S. adults needing OUD treatment (eTable 1). The probability of remaining in the "Untreated OUD state" is derived by subtracting the probabilities of MOUD initiation and Death from 1.

From MOUD-Initiated state: this health state depicts those who initiated MOUD during hospitalization (Markov cycle 1) or after discharge (Markov cycle 2 and later). Subjects in this state could continue MOUD (MOUD-Sustained), revert to Untreated OUD state, or die in the next cycle. The transition probabilities for moving to MOUD-Sustained state were obtained from the START trial follow-up data as the proportion who continued MOUD during the 30-day follow-up period among those who initiated MOUD during hospitalization in each trial arm. The probability of reverting to Untreated OUD state was derived by subtracting the probability of moving from MOUD-Initiation state to the MOUD-Sustained health state and the probability of death from 1.

© 2026 Okunogbe A et al. *JAMA Network Open*.

From MOUD-Sustained state: Those in this health state could either continue MOUD (i.e. remain in a MOUD-Sustained state), revert to untreated OUD state or die in the next cycle. The trial ends after the 30-day follow-up period, hence we are unable to derive the probabilities of remaining in MOUD-Sustained state after the follow-up period exclusively from our trial data only.

We supplemented with published estimates of longer-term retention in MOUD treatment.[4] In a prospective cohort study of adults aged 18 or older with moderate to severe OUD in the community, MOUD retention was 49% and 58% among those who initiated buprenorphine and methadone, respectively at 6-months.[4] We assume these percentages hold for the cycles after the follow-up period. We derived the probability for continuing MOUD after the follow-up period by calculating the weighted average of MOUD retention among those who continued buprenorphine and methadone in the follow-up period using the percentages for 6-month buprenorphine and methadone retention in Biondi et al (2022).[4] We do this for both the treatment and the control group.

The probability of transitioning from MOUD-Sustained state to Untreated OUD state (i.e. reverting to untreated OUD state) was derived by subtracting the probability of remaining in the MOUD-Sustained health state and the probability of dying from 1.

Death: This is the absorbing state. Participants can transition into this state from any of the other 3 health states. We use published mortality estimates for populations represented by the 3 health states: Untreated OUD, MOUD-Initiation and MOUD-Sustained. They were derived from a systematic review and meta-analysis of the effects of medication-assisted treatment on mortality among opioids users.[5] We extracted the pooled crude mortality rates (CMRs) among untreated opioid users (proxy for Untreated OUD), among opioid users that initiated MOUD (proxy for MOUD-Initiation), and among opioid users post-initiation (proxy for MOUD-Sustained). CMRs were adjusted to the one-month cycle length and converted to probabilities.

Costs

START Implementation Costs: START Implementation costs are made up of personnel costs and onboarding/training costs (Table 2). Personnel costs denote the economic cost of the time spent delivering START by the START team. The START team, comprised of an addiction medicine specialist (AMS) and care manager (CM) logged their time in registry created for the trial. This included time spent interacting with patients, time spent linking patients to follow-up care after discharge, time spent for follow-up calls within the four-weeks post discharge, and administrative time.[6] The average personnel cost per site was estimated by multiplying the mean logged time per site by hourly wage for each position using 2022 mean hourly wage data from the Bureau of Labor Statistics (BLS) for each metropolitan area included in the study. Mean hourly wage estimates were adjusted upwards by 30% to account for fringe benefits, based on the 2023 BLS Employer Costs for Employee Compensation Summary.[7] Cost estimates were then adjusted to 2023 dollars. The average personnel cost per patient across the three sites was computed as the mean logged time multiplied by the average wage across sites.

Training costs are the time costs of training and onboarding the START Team. The training and onboarding time comprised of estimated time spent by the START trial trainer, who was a psychologist involved in the study (15 hours), and trainees' time (a total of 5 hours per trainee). The training costs were computed as the total training and onboarding time for the AMS and CM at each site multiplied by the hourly wage estimates, adjusted upwards by 30% to include fringe benefits. The hourly wage of the START trial trainer was assumed to be an average of AMS wages across the three sites. The training cost per patient (Table 2) was the sum of training costs per site divided by the total number of patients randomized to the START intervention.

The implementation cost per patient (Table 1) was the sum of the personnel and training costs per patient. These costs were attributed only to patients randomized to the START intervention and only included in the first cycle.

© 2026 Okunogbe A et al. *JAMA Network Open*.

Health Outcome-Related Costs: We estimated the treatment costs associated with each health state, using the same cost estimates for the intervention and usual care arms. For each living health state, total healthcare expenditures per month were derived from published estimates of a study that tracked and reported cost of healthcare before, during, and after MOUD initiation among adults with OUD who were commercially insured or Medicare Advantage enrollees.[8] The study estimated total aggregate health care costs related to OUD and other health conditions, including both health plan and patient out-of-pocket expenditures. Expenditures were based on adjudicated claims that included medical, surgical, behavioral health, laboratory, durable medical equipment, and pharmacy claims in all inpatient and outpatient care settings and presented as mean costs per member per month.[8] Costs were converted to 2023 US dollars (Table 1).

Other Costs for Limited Societal Perspective

For the limited societal perspective, we included informal healthcare costs (from transportation and patient time costs), productivity loses due to absenteeism and presenteeism, and lost earnings due to premature death.

Informal Healthcare Costs: We included informal health care cost -transportation and patient time costs - in the analysis (eTable 2 & eTable 3). Transportation costs denote the economic cost of commuting to the place of care while patient time refers to the economic cost of the time spent receiving care. Transportation cost was calculated by multiplying the total distance traveled over a 12 month period by the standard mileage rate issued by the Internal Revenue Service (IRS) for 2023.[9] The total distance traveled for care was derived by multiplying the median distance to an Opioid treatment program (OTP) in a large central metro area by the estimated number of visits over 12 months.[10] According to guidelines in the Treatment Improvement Protocol (TIP 63) of the Substance Abuse and Mental Health Services Administration (SAMHSA)[11], doses and schedules of medications must be individualized. Hence, our estimated number of visits were calculated from a plausible treatment schedule based on national guidelines (eTable 2). We conservatively assume that all treatments take place at an Opioid Treatment Program. The transportation costs per patient are calculated separately for Methadone and Buprenorphine. We then find the weighted average of the transportation costs using the percentages in our sample that initiated Methadone and Buprenorphine.

The patient time costs were calculated by multiplying the total time spent receiving MOUD over a 12-month period by US median hourly wage from BLS (eTable 3). The total time spent was derived by multiplying the estimated time spent by the number of visits according to the assumed treatment schedule. Like the transportation costs, patient time costs were calculated separately for Methadone and Buprenorphine. We then find the weighted average of the patient time cost using the percentages in our sample that initiated Methadone and Buprenorphine.

Productivity losses from absenteeism and presenteeism and premature mortality costs: Absenteeism denotes the estimated missed work due to OUD while presenteeism denotes reduced productivity while at work due to OUD. Annual estimates of days of absenteeism and presenteeism under each living health state were derived from Henke et al (2020)[12] (eTable 4). The estimates were multiplied by BLS Household Data median hourly wage estimates for 2022 multiplied by 8 hours per workday[13] These were then divided by 12 to obtain monthly estimates. Based on employment information from the START trial, these estimates were adjusted to account for the percentage of participants that were full-time (15.10%) or part-time employed (6.50%). Premature mortality costs are lost earnings due to premature death. These costs were assigned to death state for the limited societal perspective. The premature mortality costs in a month were computed as the BLS median weekly earnings estimates multiplied by the average number of weeks in a month. The included societal costs were adjusted to 2023 US dollars.

Health Utilities

We assigned utility values to the alive health states in the Markov model: Untreated OUD, MOUD Initiated, and MOUD Sustained. Utility values were derived from Wittenberg et al. (2016),[14] which measured health-related quality of life using a standard gamble method[15] based on a US-population-representative respondent panel.

© 2026 Okunogbe A et al. *JAMA Network Open*.

Estimates were presented for 6 opioid misuse and treatment outcomes: active injection misuse; active prescription misuse; initiation stage of methadone therapy, initiation stage of buprenorphine therapy, stabilized methadone therapy, and stabilized buprenorphine therapy. For the utility in the Untreated OUD state, we use the weighted average of the utilities of active injection misuse & active prescription misuse based on the distribution of participants in the START trial with active injection versus prescription misuse at baseline.

For the utility in the MOUD-Initiated state, we use the weighted average of the utilities of initiation stage of methadone and buprenorphine treatment based on the distribution of trial participants who initiated methadone versus buprenorphine. For the utility in MOUD-Sustained state, we use the weighted average of utilities of stabilized methadone and buprenorphine therapy based on the distribution of those who continued methadone and buprenorphine therapy at follow-up. Monthly utilities for each health state were calculated by dividing utility values by 12, as presented in Table 1 of the main manuscript.

Deterministic Sensitivity Analyses

We conducted a series of 1-way sensitivity analyses to examine the implications of uncertainty on individual model parameters and to understand the key drivers of our results. Parameters tested included START implementation costs, transition probabilities, and healthcare costs under each living health state. We varied each model parameter between lower and upper bound of their feasible ranges (+/-50%, +/-15%, and +/- 30% of the base case value for costs, transition probabilities and utilities respectively) (eTable 5 & 6, Figure 1).

A one-way sensitivity analysis of special interest explored cost effectiveness if MOUD initiation in subsequent cycles for this subgroup were assumed to be as much as 48% higher than usual care, based on START trial data (eTable 7). In the base case analysis, we assumed that the probability of MOUD initiation in cycles after that the first cycle for those in the intervention group will be equal to MOUD initiation probability of those in the usual care. This is however a very conservative assumption considering that MOUD initiation was 48% higher among those who initiated MOUD in the 30-day follow-up in the intervention group compared to usual care. Hence, there is the possibility that START patients who were exposed to the intervention, but who did not initiate MOUD during hospitalization (first cycle) might have an increased probability of initiation during the 30-day follow-up period (second cycle) and subsequent cycles, relative to usual care.

Probabilistic Sensitivity Analyses

We conducted probabilistic sensitivity analysis by defining probabilistic distributions for key model parameters and running 5,000 Markov Chain Monte Carlo simulations, each using parameter values randomly selected from the distributions, until convergence was achieved.[16] We used trial or literature-based 95% CI where possible. Otherwise, we used evaluated costs, transition probabilities and utilities across +/-50%, +/-15%, and +/- 30% intervals, respectively. We used beta distributions for utilities; gamma distributions for healthcare costs; and beta and uniform distributions for transition probabilities (Table 1, Figure 2, eFigure 3 & 4).

© 2026 Okunogbe A et al. *JAMA Network Open*.

eTable 1. CHEERS (Consolidated Health Economic Evaluation Reporting System) Checklist

Section/topic	Item No	Guidance for reporting	Reported in section
**Title**			
Title	1	Identify the study as an economic evaluation and specify the interventions being compared.	Title
**Abstract**			
Abstract	2	Provide a structured summary that highlights context, key methods, results, and alternative analyses.	Abstract
**Introduction**			
Background and objectives	3	Give the context for the study, the study question, and its practical relevance for decision making in policy or practice.	Introduction
**Methods**			
Health economic analysis plan	4	Indicate whether a health economic analysis plan was developed and where available.	Methods introduction: The economic analysis is based on a randomized control trial
Study population	5	Describe characteristics of the study population (such as age range, demographics, socioeconomic, or clinical characteristics).	Methods: Setting and Participants
Setting and location	6	Provide relevant contextual information that may influence findings.	Methods: Setting and Participants
Comparators	7	Describe the interventions or strategies being compared and why chosen.	Methods: Comparators
Perspective	8	State the perspective(s) adopted by the study and why chosen.	Methods: Economic Evaluation Model Overview
Time horizon	9	State the time horizon for the study and why appropriate.	Methods: Economic Evaluation Model Overview; eMethods
Discount rate	10	Report the discount rate(s) and reason chosen.	Methods: Economic Evaluation Model Overview
Selection of outcomes	11	Describe what outcomes were used as the measure(s) of benefit(s) and harm(s).	Methods: Economic Evaluation Model Overview
Measurement of outcomes	12	Describe how outcomes used to capture benefit(s) and harm(s) were measured.	Methods: Economic Evaluation Model Overview

© 2026 Okunogbe A et al. *JAMA Network Open*.

Section/topic	Item No	Guidance for reporting	Reported in section
Valuation of outcomes	13	Describe the population and methods used to measure and value outcomes.	Methods: Economic Evaluation Model Overview; Discussion; WTP threshold based on <1 GDP per capita
Measurement and valuation of resources and costs	14	Describe how costs were valued.	Methods: Costs
Currency, price date, and conversion	15	Report the dates of the estimated resource quantities and unit costs, plus the currency and year of conversion.	Methods: Costs
Rationale and description of model	16	If modelling is used, describe in detail and why used. Report if the model is publicly available and where it can be accessed.	Methods: Economic Evaluation Model Overview; eMethods
Analytics and assumptions	17	Describe any methods for analyzing or statistically transforming data, any extrapolation methods, and approaches for validating any model used.	Methods; eMethods
Characterizing heterogeneity	18	Describe any methods used for estimating how the results of the study vary for subgroups.	Not applicable
Characterizing distributional effects	19	Describe how impacts are distributed across different individuals or adjustments made to reflect priority populations.	Not applicable
Characterizing uncertainty	20	Describe methods to characterize any sources of uncertainty in the analysis.	Methods; eMethods
Approach to engagement with patients and others affected by the study	21	Describe any approaches to engage patients or service recipients, the general public, communities, or stakeholders (such as clinicians or payers) in the design of the study.	Not applicable
**Results**			
Study parameters	22	Report all analytic inputs (such as values, ranges, references) including uncertainty or distributional assumptions.	Results; Table 1-3
Summary of main results	23	Report the mean values for the main categories of costs and outcomes of interest and summarise them in the most appropriate overall measure.	Results; Table 3
Effect of uncertainty	24	Describe how uncertainty about analytic judgments, inputs, or projections affect findings. Report the effect of choice of discount rate and time horizon, if applicable.	Results; Figures 1 & 2, eMethods

© 2026 Okunogbe A et al. *JAMA Network Open*.

Section/topic	Item No	Guidance for reporting	Reported in section
Effect of engagement with patients and others affected by the study	25	Report on any difference patient/service recipient, general public, community, or stakeholder involvement made to the approach or findings of the study	Not applicable
**Discussion**			
Study findings, limitations, generalizability, and current knowledge	26	Report key findings, limitations, ethical or equity considerations not captured, and how these could affect patients, policy, or practice.	Discussion
**Other relevant information**			
Source of funding	27	Describe how the study was funded and any role of the funder in the identification, design, conduct, and reporting of the analysis	Article Information
Conflicts of interest	28	Report authors conflicts of interest according to journal or International Committee of Medical Journal Editors requirements.	Article Information

Abbreviation: CHEERS = Consolidated Health Economic Evaluation Reporting Standards[17]

© 2026 Okunogbe A et al. *JAMA Network Open*.

eTable 2. Impact Inventory Analysis

Sector	Type of impact	Perspective		Notes
		Healthcare sector	Societal	
**Formal healthcare sector**				
Health	Health outcomes (effects)			
	Longevity effects	✓	✓	See Table 1
	Health-related quality-of-life effects	✓	✓	See Table 1
	Other health effects	✓	✓	MOUD initiation and continuation. See Table 1
	Medical costs			
	Paid for by third-party payers	✓	✓	See Table 1
	Paid for by patients out-of-pocket	✓	✓	See Table 1
	Future related medical costs (payers and patients)	✓	✓	See Table 1
	Future unrelated medical costs (payers and patients)	x	x	
**Informal healthcare sector**				
Health	Patient time costs	NA	✓	See Table 1
	Unpaid caregiver time costs	NA	x	
	Transportation costs	NA	✓	See Table 1
**Non-healthcare sectors**				
Productivity	Labor market earnings lost	NA	✓	See Table 1
	Cost of unpaid lost productivity due to illness	NA	✓	See Table 1
	Cost of uncompensated household production	NA	x	
Consumption	Future consumption unrelated to health	NA	x	
Social services	Cost of social services as part of intervention	NA	x	
Legal/ criminal justice	Number of crimes related to intervention	NA	x	
	Cost of crimes related to intervention	NA	x	
Education	Impact of intervention on educational achievement of population	NA	x	
Housing	Cost of intervention on home improvements (e.g., removing lead paint)	NA	x	
Environment	Production of toxic waste or pollution by intervention	NA	NA	
Other	Other impacts	x	x	

Impact Inventory included on recommendation from the Second Panel on Cost-Effectiveness in Health and Medicine[18].
Abbreviation: NA = not applicable, MOUD = Medications for OUD.

© 2026 Okunogbe A et al. *JAMA Network Open*.

eTable 3. Transition Probabilities for Initiating and Sustaining MOUD by Markov Cycle

**Cycle (Month)**	**Probability of MOUD initiation in index cycle**		**Probability of sustaining MOUD in the next cycle (index + 1)**		**Probability of sustaining MOUD in subsequent cycles (≥ index + 2)**	
	START	UC	START	UC	START	UC
1	57.32%	26.71%	71.43%	70.83%	54.94%	54.29%
≥2	25.12%	25.12%	71.43%	70.83%	54.94%	54.29%
Data Source	*Ober et al, 2025*[3]*; Dowell et al, 2024*[19]		*Ober et al, 2025*[3]		*Ober et al, 2025*[3]*; Biondi et al, 2022*[4]	

Abbreviations: MOUD = Medications for OUD.

eTable 4. Transportation Costs

**Treatment Schedule**				
	**Number of visits per week**	**Total Visits**	**Transportation distance (miles) – round trip**	**Transportation cost ($)**
**Methadone**				
1st 12 months	7	336	2688	1760.64
Subtotal		336	2688	1760.64
**Buprenorphine**				
1st week	first 3 days until stabilization	3	24	15.72
Week 2-4	once a week	3	24	15.72
2nd month	biweekly	2	16	10.48
3rd-5th month	once a month	3	24	15.72
6th-12th month	once in 6 months	1	8	5.24
Subtotal		12	96	62.88
Patient Transportation Cost (Methadone and Buprenorphine) –Annual				$1,131.40
Patient Transportation Cost (Methadone and Buprenorphine) –Monthly				$94.28

Cost derived as total distance multiplied by IRS reimbursement mileage rate. The median driving distance to an OTP in a large central metro is 4.0 miles. IRS reimbursement rate for 2023 is $0.655. Annual and monthly patient transportation cost are the weighted averages of costs for those on methadone and buprenorphine. Approximately 63% of our sample initiated methadone and 37% initiated buprenorphine.

© 2026 Okunogbe A et al. *JAMA Network Open*.

eTable 5. Patient Time Costs

Time spent at OTP and number of visits by treatment schedule				
**Methadone**				
	Number of visits	Time per visit (hrs)	Total time (hrs)	Time costs ($)
Day 1	1	4	4	109.75
Day 2-7	6	2	12	329.24
Week 2- 4	21	1	21	576.17
Months 2-12	308	0.25	77	2,112.64
			114	3,127.80
**Buprenorphine**				
	Number of visits	Time per visit (hrs)	Total time (hrs)	Time costs ($)
Day 1 in Week 1	1	3	3	82.31
Day 2-3 in Week 1	2	1.5	3	82.31
Week 2- Week 4	3	0.5	1.5	41.16
Month 2-6	5	0.5	2.5	68.59
Month 7-12	1	0.5	0.5	13.72
			10.5	288.09
Patient Time Cost (Methadone and Buprenorphine) - Annual				$2075.32
Patient Time Cost (Methadone and Buprenorphine) - Monthly				$172.94

Patient time cost derived as total time spent receiving MOUD multiplied by median hourly wages. The median hourly wage is $27.45 adjusted to 2023 USD. Annual and monthly patient time costs are the weighted averages of costs for those on methadone and buprenorphine. Approximately 63% of our sample initiated methadone and 37% initiated buprenorphine. Abbreviations: OUD = Opioid Use Disorder, MOUD = Medications for OUD, OTP = Opioid Treatment Program.

eTable 6. Productivity Losses due to Absenteeism and Presenteeism Associated with OUD

Health State	Annual Additional Absenteeism Days[12]	Annual Additional Lost-at work productivity (Presenteeism) Days (Annually)[12]	Annual Absenteeism and Presenteeism Cost	Monthly Absenteeism and Presenteeism Cost
Untreated OUD	0.67	14.1	$ 594.90	$ 49.57
MOUD- Initiated	0.32	1.9	$ 89.42	$ 7.45
MOUD- Sustained	0.32	1.9	$ 89.42	$ 7.45

*We assume additional absenteeism and presenteeism will be the same under MOUD-initiated and MOUD-sustained state. Annual Absenteeism and Presenteeism cost was calculated as the summation of additional absenteeism and presentism days due to OUD multiplied by median daily wages and percent of OUD employed. The percentage of participants that were full-time was 15.10% and percentage that were part-time was 6.50%. We assume those partially employed earn 50% of full wages. Abbreviations: OUD = Opioid Use Disorder, MOUD = Medications for OUD.

© 2026 Okunogbe A et al. *JAMA Network Open*.

eTable 7: Incremental Cost-Effectiveness of the Substance Use Treatment and Recovery Team" (START) Intervention (2023 US Dollars)-- Key Scenario*

Strategies	Net Cost per Patient, $ (95% UI)	Incremental Net Cost per Patient, $ (95% UI)	Quality-Adjusted Life Years (QALYs) per Patient (95% UI)	Incremental QALYs per Patient (95% UI)
Health Sector Perspective				
START	49,611 (49,286 -49,710)	-432 (-530 – -393)	0.657 (0.657 – 0.657)	0.0213 (0.0210 - 0.0215)
Usual Care	50,044 (49,713 -50,206)		0.636 (0.635 - 0.636)	
Limited Societal Perspective				
START	52,568 (52,394 - 52,824)	-237 (-340 - -205)	0.657 (0.657 – 0.657)	0.0213 (0.0213-0.0218)
Usual Care	52,806 (52,634 – 53,130)		0.636 (0.636 - 0.636)	

*Scenario where probability of MOUD initiation for START is 48 percent higher than probability of MOUD initiation under Usual care in subsequent cycles after the 1st cycle.
Abbreviations: QALYs = quality-adjusted life-years, UI = Uncertainty Interval, MOUD = Medications for OUD.

© 2026 Okunogbe A et al. *JAMA Network Open*.

eTable 8. Deterministic Sensitivity Analyses (Health Sector Perspective)

	Parameter Value/Range			ICER (95% UI)	
**Parameter**	***Baseline Value***	***Lower Bound***	***Upper Bound***	***Lower Bound***	***Upper Bound***
Monthly average healthcare cost when OUD is untreated ($)	4,184	2,092	6,276	-185,786	217,286
Utility of Untreated OUD	0.05	0.03	0.06	8,034	398,390
Monthly average healthcare cost when MOUD is initiated ($)	4,149	2,075	6,224	-97,929	129,429
Probability of continuing MOUD among those who initiated in START	0.71	0.61	0.82	-16,739	139,754
Probability of initiating MOUD for START (1st cycle)	0.57	0.49	0.66	-8,364	133,277
Probability of initiating MOUD for START (2nd-12th cycles)	0.25	0.21	0.29		
Probability of retention in sustained MOUD for START	0.55	0.47	0.63	-20,947	109,849
Monthly average healthcare cost when MOUD is sustained ($)	2,886	1,443	4,329	-48,824	80,323
Probability of retention in sustained MOUD for Usual Care	0.54	0.46	0.62	-11,686	115,127
Probability of continuing MOUD among those who initiated in Usual Care	0.71	0.60	0.81	-14,933	95,534
Probability of initiating MOUD for Usual Care (1st Cycle)	0.27	0.23	0.31	-9,411	93,078
Probability of initiating MOUD for Usual Care (2nd-12th cycles)	0.25	0.21	0.29		
Utility of MOUD-Sustained/Continued	0.06	0.04	0.08	8,495	107,941
START ACS Implementation cost ($)	640	320	960	-15,409	46,908
Utility of MOUD-Initiated	0.05	0.04	0.07	11,199	26,531
Probability of death among untreated OUD	0.0041	0.0035	0.00	14,397	17,042
Probability of death among those who initiate MOUD	0.0012	0.0010	0.00	15,588	15,911
Probability of death among those who continue MOUD	0.0005	0.0004	0.00	15,662	15,837

Negative ICERs denotes that the average cost of START is lower than the average cost of Usual care. Parameters listed based on the ranges with the largest impact on ICER as shown in Figure 1. Model parameters were varied between lower and upper bound of their feasible ranges (+/-50%, +/-15%, and +/- 30% of the base case value for costs, transition probabilities and utilities respectively). Abbreviations: START = Substance Use Treatment and Recovery Team, OUD = Opioid Use Disorder, MOUD = Medication for OUD, ACS = Addiction Consultation Service, ICER = Incremental Cost-Effectiveness Ratio, UI = Uncertainty Interval.

© 2026 Okunogbe A et al. *JAMA Network Open*.

eTable 9. Deterministic Sensitivity Analyses (Limited Societal Perspective)

Parameter	Parameter Values/Range			ICER (95% UI)	
	Baseline Value	***Lower Bound***	***Upper Bound***	***Lower Bound***	***Upper Bound***
Utility of Untreated OUD	0.05	0.03	0.06	10,671	529,198
Monthly average healthcare cost when OUD is untreated ($)	4,184	2,092	6,276	-180,615	222,457
Monthly average healthcare cost when MOUD is initiated ($)	4,149	2,075	6,224	-92,758	134,600
Probability of continuing MOUD among those who initiated in START	0.71	0.61	0.82	-11,187	143,384
Utility of MOUD-Sustained/Continued	0.06	0.04	0.08	11,284	143,383
Probability of initiating MOUD for START (1st cycle)	0.57	0.49	0.66	-1,400	129,776
Probability of initiating MOUD for START (2nd-12th cycles)	0.25	0.21	0.29		
Monthly average healthcare cost when MOUD is sustained ($)	2,886	1,443	4,329	-43,653	85,495
Probability of retention in sustained MOUD for START	0.55	0.47	0.63	-14,905	112,965
Probability of retention in sustained MOUD for Usual Care	0.54	0.46	0.62	-5,753	117,369
Probability of continuing MOUD among those who initiated in Usual Care	0.71	0.60	0.81	-9,182	99,247
Probability of initiating MOUD for Usual Care (1st Cycle)	0.27	0.23	0.31	-2,168	91,981
Probability of initiating MOUD for Usual Care (2nd-12th cycles)	0.25	0.21	0.29		
START ACS Implementation cost ($)	640	320	960	-10,237	52,079
Utility of MOUD-Initiated	0.05	0.04	0.07	14,876	35,243
Monthly average patient time cost ($)	173	86	259	14,889	26,953
Premature mortality costs	4,756	2,378	7,134	15,600	26,242
Monthly average transportation cost ($)	94	24	165	15,988	25,854
Absenteeism cost when untreated	50	25	74	19,248	22,594
Probability of death among untreated OUD	0.0041	0.0035	0.0047	20,245	21,629
Absenteeism cost when in treatment	7	4	11	20,661	21,181
Probability of death among those who initiate MOUD	0.0012	0.001	0.0014	20,828	21,015
Probability of death among those who continue MOUD	0.0005	0.0004	0.0005	20,871	20,971

Negative ICERs denotes that the average cost of START is lower than the average cost of Usual care. Parameters listed based on the ranges with the largest impact on ICER as shown in Figure 1. Model parameters were varied between lower and upper bound of their feasible ranges (+/-50%, +/-15%, and +/- 30% of the base case value for costs, transition probabilities and utilities respectively). Abbreviations: START = Substance Use Treatment and Recovery Team, OUD = Opioid Use Disorder, MOUD = Medication for OUD, ACS = Addiction Consultation Service, ICER = Incremental Cost-Effectiveness Ratio, UI = Uncertainty Interval.

© 2026 Okunogbe A et al. *JAMA Network Open*.

eFigure 1. State Transition Diagram

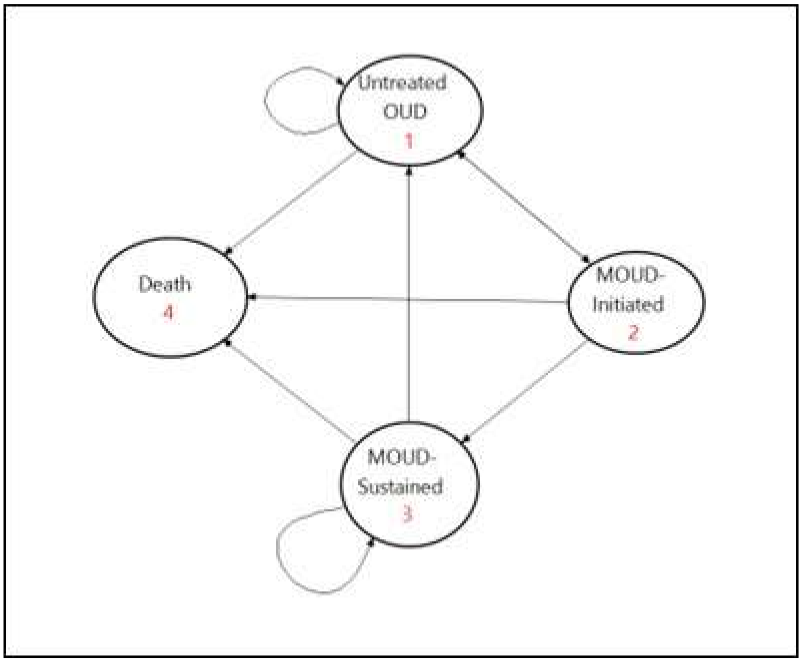

Abbreviation: OUD = Opioid Use Disorder, MOUD = Medications for OUD

eFigure 2. Markov Model Diagram

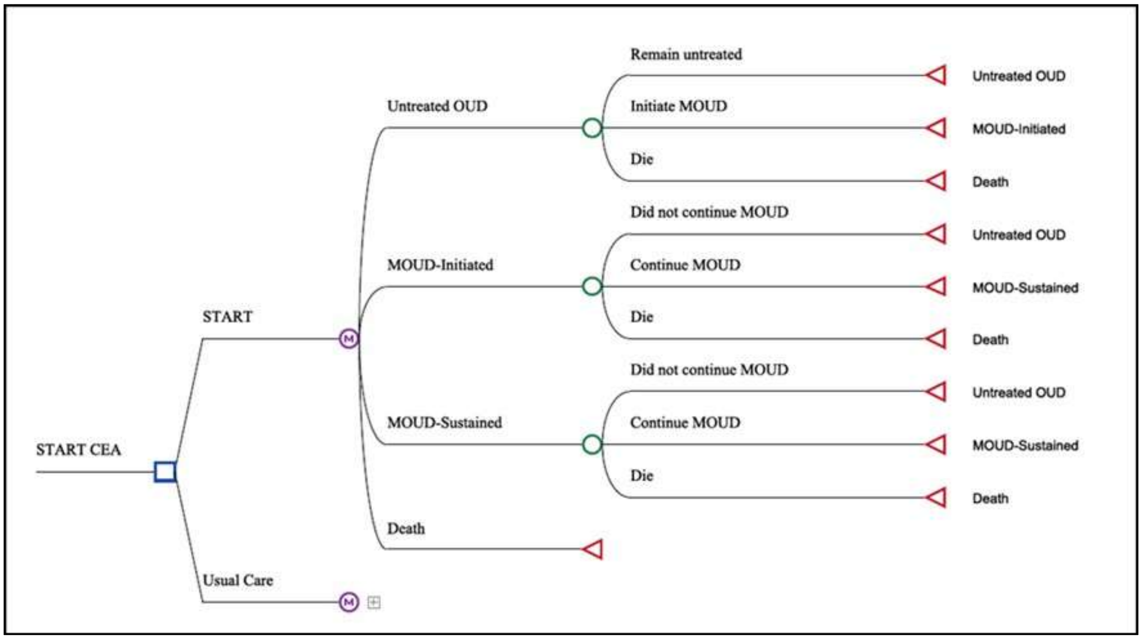

*Usual Care has the same branches as START.

© 2026 Okunogbe A et al. *JAMA Network Open*.

eFigure 3. Probabilistic Sensitivity Analysis Scatter Plot: Incremental Cost-Effectiveness from the Health Sector Perspective (based on 5,000 simulations)-Key Scenario*

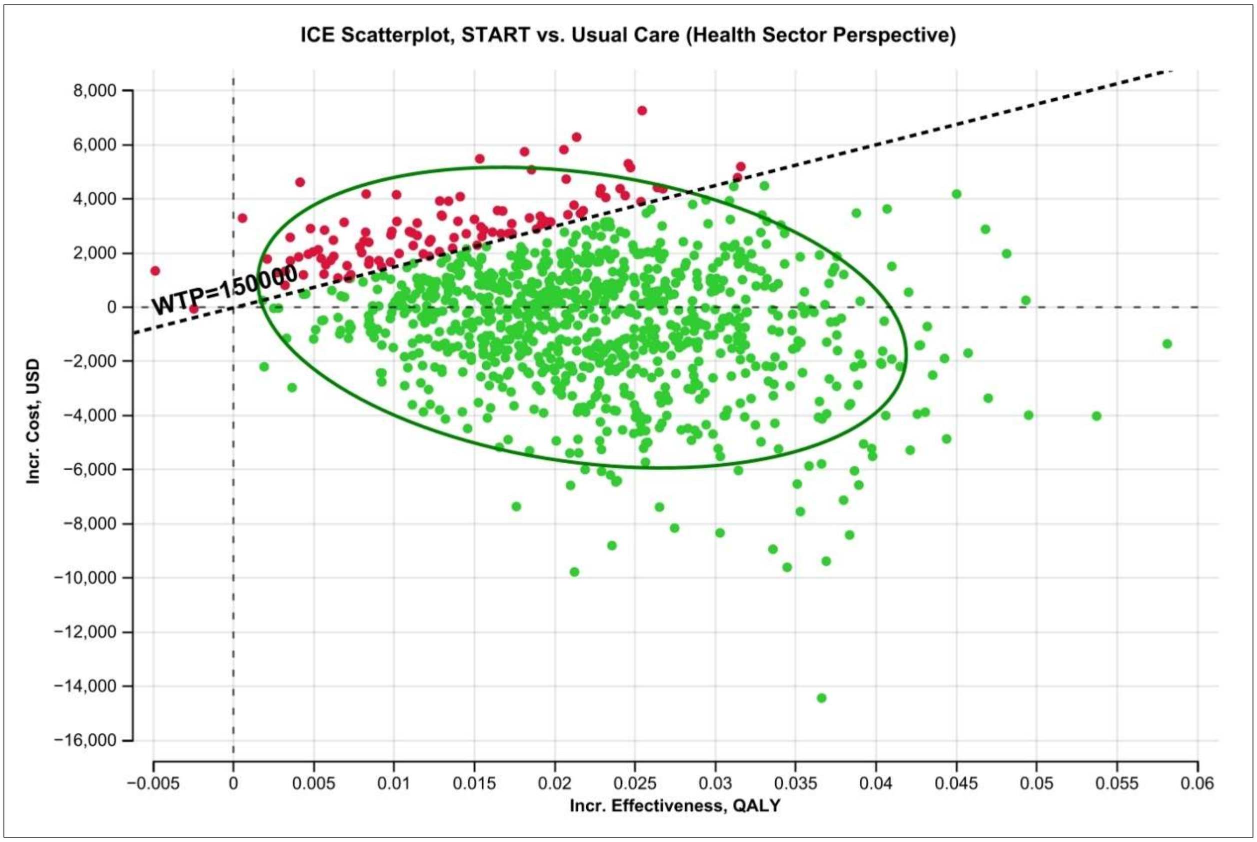

*Scenario where probability of START initiation is 48 percent higher than probability under usual care in subsequent cycles after the 1st cycle. START is the optimal strategy in 90% of simulations. Abbreviations: ICE = Incremental Cost- Effectiveness, QALY = Quality-Adjusted Life Years, WTP = Willingness-to-pay, Incr. = Incremental, USD = US Dollars.

© 2026 Okunogbe A et al. *JAMA Network Open*.

eFigure 4. Probabilistic Sensitivity Analysis Scatter Plot: Incremental Cost-Effectiveness from the Limited Societal Perspective (based on 5,000 simulations) - Key Scenario*

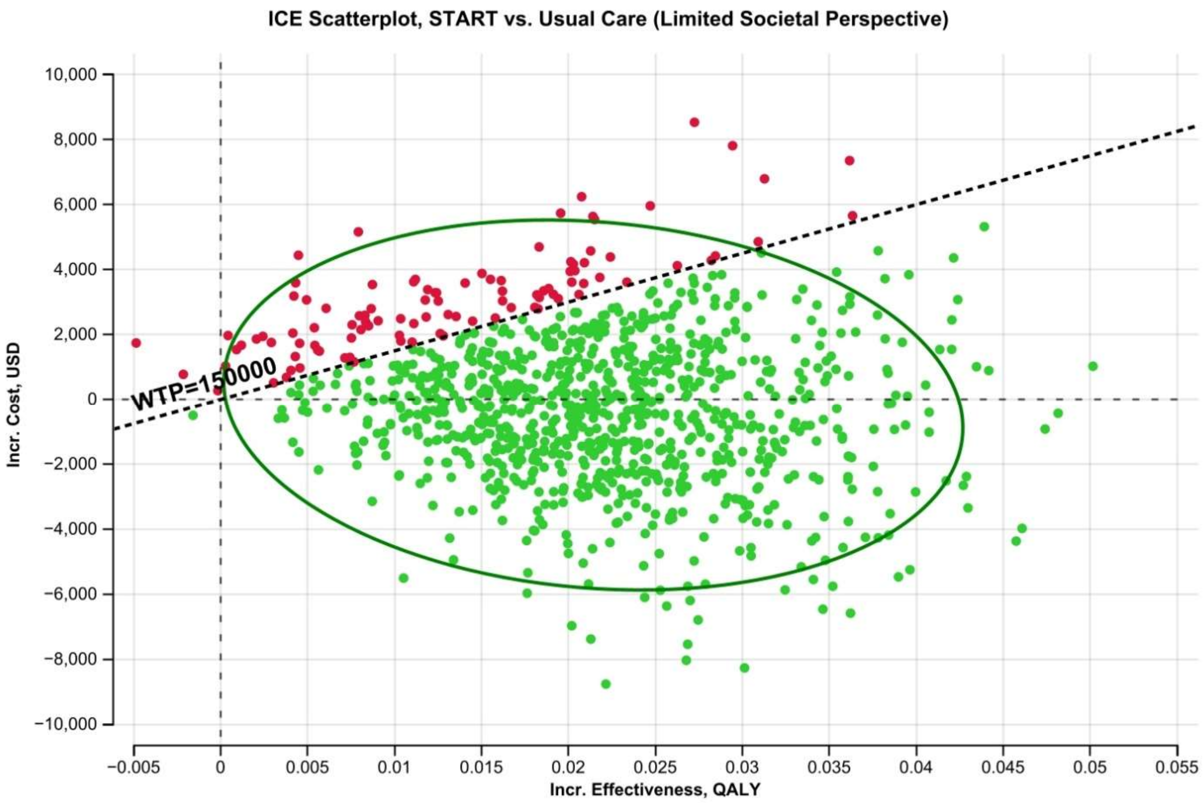

*Scenario where probability of START initiation is 48 percent higher than probability under usual care in subsequent cycles after the 1st cycle. START is the optimal strategy in 89% of simulations. Abbreviations: ICE = Incremental Cost- Effectiveness, QALY = Quality-Adjusted Life Years, WTP = Willingness-to-pay, Incr. = Incremental, USD = US Dollars.

© 2026 Okunogbe A et al. *JAMA Network Open*.

eFigure 5. ICER Estimates by Time Horizon

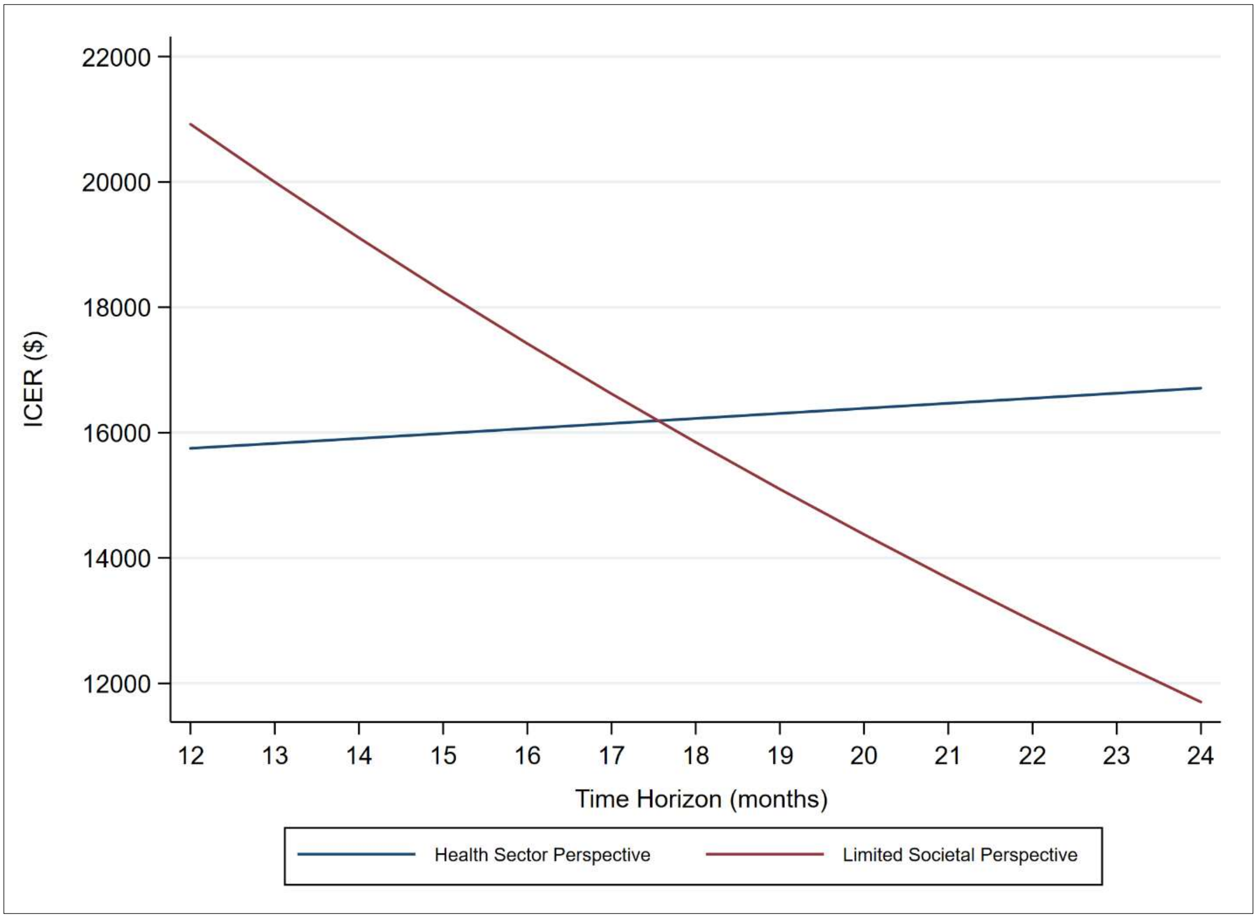

ICER at different time horizons using the same set of assumptions from 12 months to 24 months.
Abbreviation: ICER = Incremental Cost-Effectiveness Ratio

© 2026 Okunogbe A et al. *JAMA Network Open*.

References

1. TreeAge Pro 2025, R1.

2. StataCorp.

3. Ober AJ, Murray-Krezan C, Page K, Friedmann PD, Anderson J, Osilla KC, Ryzewicz S, Huerta S, Mazer MW, Hoskinson RA, Garvey R, Peltz A, Watkins KE, Nuckols T, IsHak WW, Mariano LT, Danovitch I. Hospital Addiction Consultation Service and Opioid Use Disorder Treatment: The START Randomized Clinical Trial. *JAMA Internal Medicine*. Published online April 7, 2025. doi:10.1001/jamainternmed.2024.8586

4. Biondi BE, Vander Wyk B, Schlossberg EF, Shaw A, Springer SA. Factors associated with retention on medications for opioid use disorder among a cohort of adults seeking treatment in the community. *Addict Sci Clin Pract*. 2022;17:15. doi:10.1186/s13722-022-00299-1

5. Ma J, Bao YP, Wang RJ, Su MF, Liu MX, Li JQ, Degenhardt L, Farrell M, Blow FC, Ilgen M, Shi J, Lu L. Effects of medication-assisted treatment on mortality among opioids users: a systematic review and meta-analysis. *Mol Psychiatry*. 2019;24(12):1868-1883. doi:10.1038/s41380-018-0094-5

6. Osilla KC, Ober AJ, Leamon I, Messineo G, Danovitch I. *Substance Use Treatment and Recovery Team Addiction Consultation Service (START ACS): An Addiction Consultation Service for Opioid Use Disorder*. RAND Corporation; 2025. Accessed April 30, 2025. https://www.rand.org/pubs/tools/TLA3791-1.html

7. Bureau of Labor Statistics. Employer Costs for Employee Compensation – March 2023.

8. Larochelle MR, Wakeman SE, Ameli O, Chaisson CE, McPheeters JT, Crown WH, Azocar F, Sanghavi DM. Relative Cost Differences of Initial Treatment Strategies for Newly Diagnosed Opioid Use Disorder: A Cohort Study. *Medical Care*. 2020;58(10):919. doi:10.1097/MLR.0000000000001394

9. IRS issues standard mileage rates for 2023; business use increases 3 cents per mile | Internal Revenue Service. Accessed July 7, 2025. https://www.irs.gov/newsroom/irs-issues-standard-mileage-rates-for-2023-business-use-increases-3-cents-per-mile

10. Kleinman RA. Comparison of Driving Times to Opioid Treatment Programs and Pharmacies in the US. *JAMA Psychiatry*. 2020;77(11):1-9. doi:10.1001/jamapsychiatry.2020.1624

11. TIP 63: Medications for Opioid Use Disorder | SAMHSA Library. Accessed July 7, 2025. https://library.samhsa.gov/product/tip-63-medications-opioid-use-disorder/pep21-02-01-002

12. Henke RM, Ellsworth D, Wier L, Snowdon J. Opioid Use Disorder and Employee Work Presenteeism, Absences, and Health Care Costs. *Journal of Occupational and Environmental Medicine*. 2020;62(5):344. doi:10.1097/JOM.0000000000001830

13. Bureau of Labor Statistics. 2022 Annual Averages - Household Data - Tables from Employment and Earnings. Accessed April 4, 2025. https://www.bls.gov/cps/cps_aa2022.htm

14. Wittenberg E, Bray JW, Aden B, Gebremariam A, Nosyk B, Schackman BR. Measuring benefits of opioid misuse treatment for economic evaluation: health-related quality of life of opioid-dependent individuals and their spouses as assessed by a sample of the US population. *Addiction*. 2016;111(4):675-684. doi:10.1111/add.13219

© 2026 Okunogbe A et al. *JAMA Network Open*.

15. Hunink MGM, Weinstein MC, Wittenberg E, Drummond MF, Pliskin JS, Wong JB, Glasziou PP. *Decision Making in Health and Medicine: Integrating Evidence and Values*. Cambridge University Press; 2014.

16. Hatswell AJ, Bullement A, Briggs A, Paulden M, Stevenson MD. Probabilistic Sensitivity Analysis in Cost-Effectiveness Models: Determining Model Convergence in Cohort Models. *PharmacoEconomics*. 2018;36(12):1421-1426. doi:10.1007/s40273-018-0697-3

17. Husereau D, Drummond M, Augustovski F, de Bekker-Grob E, Briggs AH, Carswell C, Caulley L, Chaiyakunapruk N, Greenberg D, Loder E, Mauskopf J, Mullins CD, Petrou S, Pwu RF, Staniszewska S. Consolidated Health Economic Evaluation Reporting Standards 2022 (CHEERS 2022) Statement: Updated Reporting Guidance for Health Economic Evaluations. *Pharmacoeconomics*. 2022;40(6):601-609. doi:10.1007/s40273-021-01112-8

18. Sanders GD, Neumann PJ, Basu A, Brock DW, Feeny D, Krahn M, Kuntz KM, Meltzer DO, Owens DK, Prosser LA, Salomon JA, Sculpher MJ, Trikalinos TA, Russell LB, Siegel JE, Ganiats TG. Recommendations for Conduct, Methodological Practices, and Reporting of Cost-effectiveness Analyses: Second Panel on Cost-Effectiveness in Health and Medicine. *JAMA*. 2016;316(10):1093-1103. doi:10.1001/jama.2016.12195

19. Dowell D. Treatment for Opioid Use Disorder: Population Estimates — United States, 2022. *MMWR Morb Mortal Wkly Rep*. 2024;73. doi:10.15585/mmwr.mm7325a1

© 2026 Okunogbe A et al. *JAMA Network Open*.
