# Supplementary material for: Cost-Effectiveness of the START Hospital Addiction Consultation Service for Opioid Use Disorder Treatment
Source: JAMA Netw Open. 2026 May 7;9(5):e2611324. doi: 10.1001/jamanetworkopen.2026.11324 (PMC13153993; doi:10.1001/jamanetworkopen.2026.11324)
Supplement: Supplement 2. — Data Sharing Statement [file jamanetwopen-e2611324-s002.pdf]

Data Sharing Statement

Okunogbe. Cost-Effectiveness of the START Hospital Addiction Consultation Service for Opioid Use Disorder Treatment. *JAMA Netw Open*. Published May 07, 2026. doi:10.1001/jamanetworkopen.2026.11324

Data
Data available: No

Additional Information
Explanation for why data not available: The datasets generated and analyzed during this study are not publicly available due to the sensitive nature of the data. They can be made available from the corresponding author on reasonable request and with the execution of appropriate Data Use Agreements.
